# The gut microbiome of hooded cranes (*Grus monacha*) wintering at Shengjin Lake, China

**DOI:** 10.1002/mbo3.447

**Published:** 2017-01-26

**Authors:** Guanghong Zhao, Lizhi Zhou, Yuanqiu Dong, Yuanyuan Cheng, Yunwei Song

**Affiliations:** ^1^ School of Resources and Environmental EngineeringAnhui UniversityHefeiChina; ^2^ Shengjin Lake National Nature Reserve of Anhui ProvinceChizhouChina

**Keywords:** 16S rRNA gene, gut microbiome, Illumina high‐throughput sequencing, wintering hooded cranes

## Abstract

Gut microbes of animals play critical roles in processes such as digestion and immunity. Therefore, identifying gut microbes will shed light on understanding the annual life of animal species, particularly those that are threatened or endangered. In the present study, we conducted nucleotide sequence analyses of the 16S rRNA genes of gut microbiome of the hooded cranes (*Grus monacha*) wintering at Shengjin Lake, China, by Illumina high‐throughput sequencing technology. We acquired 503,398 high‐quality sequences and 785 operational taxonomic units (OTUs) from 15 fecal samples from different cranes, representing 22 phyla that were dominated by Firmicutes, Proteobacteria, and Actinobacteria. A total of 305 genera were identified that were dominated by *Clostridium*,* Lysinibacillus*, and *Enterobacter*. The core gut microbiome comprised 26 genera, including many probiotic species such as *Clostridium*,* Bacillus*,* Cellulosilyticum*, and *Cellulomonas* that could catabolize cellulose. The findings reported here contribute to our knowledge of the microbiology of hooded cranes and will likely advance efforts to protect waterbirds that inhabit Shengjin Lake Reserve during winter.

## 1 INTRODUCTION

Gut microbes play a crucial role in the physiology of their vertebrate hosts by contributing to functions such as the development of intestinal morphology, nutrition, and immunity (Leser & Mølbak, 2009; Young, 2012). Studies of the gut microbiota of avian species have focused on the effects of specific bacteria or bacterial pathogens (Collins et al., 2002; Marois et al., 2002). Recent studies of the gut microbial communities of birds are commonplace and typically employ high‐throughput sequencing technologies and bioinformatics tools to identify microbial species and their activities. An increasing number of studies have characterized the gastrointestinal microbiota of avian species, including turkeys (*Meleagris gallopavo*) and the kakapo (*Strigops habroptilus*), showing that the gut microbes of birds play an important role in digestive physiology and immunity (Lu & Domingo, 2008; Waite et al., 2012).

Numerous studies show that the genes of parasitifer as well as environmental factors alter the composition of intestinal microflora (Stanley et al., 2013). The health of the parasitifer depends on microbial metabolism, and the microbial composition of the gut depends on the dietary habits of the parasitifer. The gastrointestinal microbiota of the parasitifer may reflect their particular lifestyles. Links between the structure of microbial communities and the diet of the parasitifer have been demonstrated in humans and animals; the intestinal microflora varies among humans who engage in different lifestyles (Zhang et al., 2015). A study of the giant panda found that the intestinal microbial composition is determined by its feeding habits, which includes large numbers of clostridial species that degrade cellulose (Zhu et al., 2011). Similarly, the gastrointestinal microbiota of avian species may reflect a particular lifestyle. For example, the hoatzin (*Opisthocomus hoazin*) feeds mostly on leaves and carries out foregut fermentation; thus, the foregut microbiota is dominated by Bacteroidetes, Firmicutes, and Proteobacteria, similar to the gut microbiota of a bovine ruminant (Domínguez‐Bello et al., 1994; Garcia‐Amado et al., 2003; Godoyvitorino et al., 2012).

Despite the presence of numerous probiotics within the avian intestinal microflora that are associated with the avian diet, numerous studies found that potential pathogens are present as well. Identification of the latter is likely of great value to contribute a better understanding of avian health, particularly for the benefit of basic research and the conservation of endangered species. For example, the identification of the intestinal microbial composition of the kakapo detects pathogens before their numbers expand to levels that induce disease (Waite et al., 2012). Moreover, studies of the intestinal microflora of waterfowl, such as gulls (*Larus audouinii*), black‐winged stilts (*Himantopus himantopus*) (Camarda et al., 2006; Grond et al., 2014; Santos et al., 2012), identified numerous potential human pathogens.

The hooded crane (*Grus monacha*) is a large migratory water bird that breeds in the eastern part of Siberia and China's Lesser Khingan mountains, and winters in Japan, South Korea, the middle and lower reaches of the Yangtze River in China (Jiao et al., 2014). The hooded crane was designated a vulnerable species in the IUCN (International Union for Conservation of Nature and Natural Resources) Red List and was classified as a national grade 1 protected bird in China, and its global population is approximately 11,600 (Bird Life International, 2012). Recently, due to many human activities, such as lakes aquaculture, tourism development, and water conservancy construction, the existing wetland aquatic resources, a number of floating‐leaved plants, submerged plants were destroyed, and a sharp decline in the number of benthic fauna was found (Chen et al., 2011). Hooded crane wintering foraging habitat has been destroyed, the main food resources of plants such as *Vallisneria natans* and *Potamogeton malaianus* for wintering populations are declining (Fox et al., 2011; Liu et al., 2001; Xu et al., 2008), increasing the survival pressure of wintering hooded cranes. Because of this, researchers are paying increased attention to the demographic and behavioral ecology of migrating birds, particularly to changes in their food supplies (Chen et al., 2011; Zhao et al., 2013; Zhou et al., 2010). However, the gut microbiome of hooded cranes is unknown. Because the gut microbiome serves as an important indicator of lifestyle and health, it is important to characterize the gut microbiome of hooded cranes to contribute to efforts to conserve this endangered species. Therefore, we use multiplex pyrosequencing to identify the species that populate the gut microbiome of wintering hooded cranes in this study.

## 2 MATERIALS AND METHODS

### 2.1 Fecal sample collection

We used noninvasive techniques (Darimont et al., 2008) to collect 15 fecal samples from hooded cranes wintering at Shengjin Lake (south bank of the Yangtze River, Hefei, China; 30.25°–30.50°N, 116.92°–117.25°E) during the wintering period, and make sure that the fecal samples were only from the cranes. Before the samples were collected, we used binoculars or telescopes to observe the foraging areas of the cranes, select large groups with more than 50 individuals, and insure the absence of other cranes or geese within the range of about 50 m in the foraging areas of the cranes. After the cranes vacated their foraging grounds, samples were rapidly collected into sterile 50 ml centrifuge tubes. It is identified as the foraging areas of the cranes according to the footprints or foraging pits left by the cranes. To minimize possible contamination from the ground, we only collected the upper layer of a fecal ball. The fecal samples were transported to the laboratory on ice and stored at −80°C.

### 2.2 DNA extraction and PCR amplification

DNA was extracted from fecal samples according to the manufacturer's instructions by the QIAamp Fast DNA Stool Mini Kit (Qiagen, Germany). We conducted tag‐pyrosequencing analysis of the V3–V4 region of 16S rRNA gene to identify intestinal bacteria. We amplified this region using the broadly conserved primers, 338F (5′‐GGACTACHVGGGTWTCTAAT‐3′) and 806R (5′‐GGACTACHVGGGTWTCTAAT‐3′) (Dennis et al., 2013), containing the A and B sequencing adaptors. Different barcode sequences were used to tag these primers to analyze multiple samples. Each sample reaction mixture (20 μl) contained 0.5 μl of 5 U/μl Easy Taq DNA polymerase, 2 μl of 10 × Easy Taq buffer, 2 μl of 0.25 mmol/L dNTPs, 0.2 μmol/L of each primer, 10 ng of template DNA, and deionized ultrapure water (to 20 μl). An Applied Biosystems GeneAmp PCR System 9700 was used to amplify the DNA samples as follows: initial denaturation at 94°C (3 min) followed by 27 cycles at 95°C (30 s), 55°C (30 s), and 72°C (45 s), and final extension at 72°C for 10 min. We used a 2% (w/v) Tris‐Boric acid‐EDTA(TBE) agarose gel to assess the quality of the amplicons.

### 2.3 Illumina MiSeq sequencing

The amplicons were purified using a MiniElute PCR purification kit (Axygen) and quantified using the Applied Biosystems GeneAmp PCR System 9700. The PCR products were pooled at equal concentrations and pyrosequencing was performed using an Illumina Miseq System at Majorbio Bio‐pharm Technology Co., Ltd. (Shanghai, China). NCBI SRA database accession numbers are SAMN05207073, SAMN05207074, SAMN05207075, SAMN05207076, SAMN05207077, SAMN05207078, SAMN05207079, SAMN05211008, SAMN05211009, SAMN05211011, SAMN05211012, SAMN05211013, SAMN05211014, SAMN05211015, and SAMN05211016.

### 2.4 Data processing and analysis

All sequences acquired using the Illumina–MiSeq were saved in the raw fastq files. Initial processing of the raw dataset included screening to remove short and low‐quality reads; only high‐quality sequences without primer sequences were retained. Using USEARCH (version 7.1 http://drive5.com/uparse/), these high‐quality sequences were clustered into operational taxonomic units (OTUs) at a 97% identity threshold. The taxonomic classification of OTUs was performed using the Ribosomal Database Project Classifier with the QIIME bioinformatics pipeline (http://qiime.org/scripts/assign_taxonomy.html) to determine the community composition of each sample at each taxonomic level (Wang et al., 2007) at a 70% confidence level. Rarefaction curves and alpha diversity calculations were based on OTUs with >97% identity. Rarefaction analysis and alpha‐diversity indices (abundance‐based coverage estimation (ACE), Chao1, Shannon and Simpson) were calculated using Mothur. R language tools were used to generate rarefaction curves (Amato et al., 2013) and Shannon–Wiener curves (Wang et al., 2012). Heatmaps were generated using the R package (Jami et al., 2013). The core fecal microbiome of the hooded cranes was assigned if it comprised >90% of samples and represented ≤0.1% of the reads.

## 3 RESULTS

### 3.1 Barcoded 16S pyrosequencing

We acquired 503,398 valid reads from the 15 fecal samples, and among them, 96.5% (average) were classified as bacterial phyla, 3.5% could not be classified, and >90% were assigned to the levels of phylum, class, and order. The average percentages of reads assigned to bacteria at the family, genus, and species levels were 82.8%, 78.7%, and 20.8%, respectively, and 22 phyla and 305 genera were identified from the 15 fecal samples.

**Figure 1:**
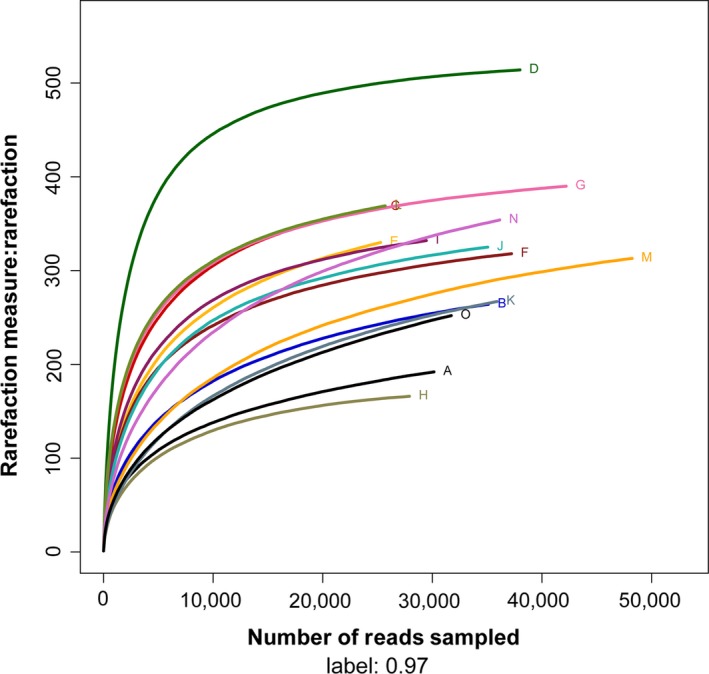
The rarefaction curves of 15 samples

**Figure 2:**
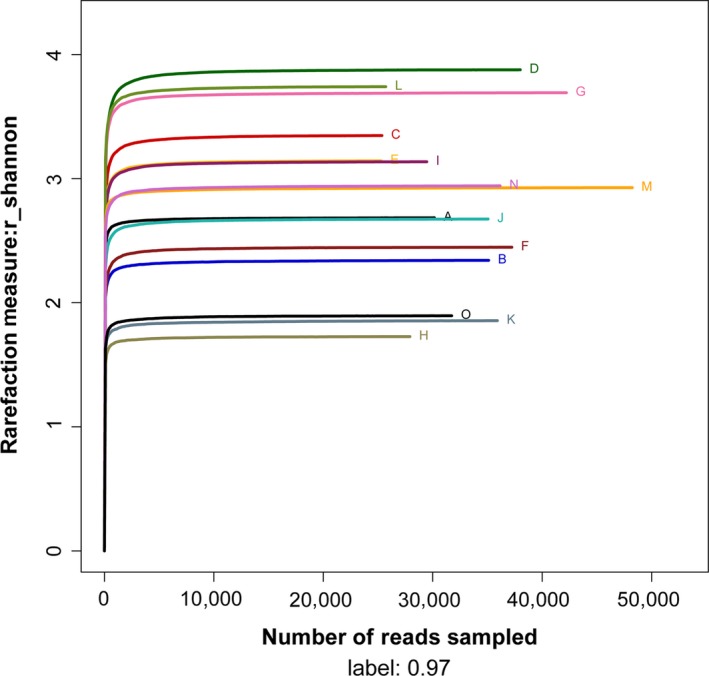
The Shannon–Wiener curves of 15 samples

Alpha diversity curves of 15 samples determined using multiplex pyrosequencing with OTUs >97% identity. The rarefaction curves of each sample are shown in Figure 1 and the Shannon–Wiener curves of each sample are shown in Figure 2. The rarefaction curves for the 15 samples plateaued at 20,000 reads, indicating that the composition of the fecal microbiota was similar to that measured in this study. Table 1 indicates the diversity index of each sample. Reads ranged from 25,292 to 48,132, which accounted for the number of OTUs, ACE, Chao1, Shannon–Wiener index, and Simpson diversity index. The number of OTUs detected per sample ranged from 166 to 514 using a cutoff of 97% identity for species‐level distinctions. Furthermore, the Shannon–Wiener Index reflected the trends of detected and estimated richness, for example, diversities of samples D and H were highest and lowest, respectively.

**Table 1:** Diversity and richness of the gut microbial communities of the wintering hooded cranes

Samples	Reads	OTUs	ACE	Chao1	Coverage	Shannon	Simpson
A	30,152	192	247	243	.998275	2.68	.1279
B	35,109	264	313	301	.998348	2.34	.2159
C	25,356	368	404	401	.997831	3.35	.1151
D	37,999	514	525	523	.999289	3.88	.0994
E	25,279	330	384	415	.997112	3.14	.1002
F	37,227	318	351	364	.998630	2.45	.2432
G	42,212	390	411	417	.999029	3.69	.0662
H	27,913	166	181	176	.999104	1.73	.3894
I	29,452	332	359	365	.998370	3.14	.112
J	35,053	325	358	368	.998459	2.67	.2044
K	35,904	267	346	336	.997772	1.86	.3461
L	25,703	369	404	414	.997821	3.74	.058
M	48,235	313	378	374	.998404	2.93	.0886
N	36,148	354	430	452	.997483	2.94	.1133
O	31,728	252	441	360	.997163	1.9	.3181

OTUs, operational taxonomic units.

### 3.2 Microbial composition of fecal samples

**Figure 3:**
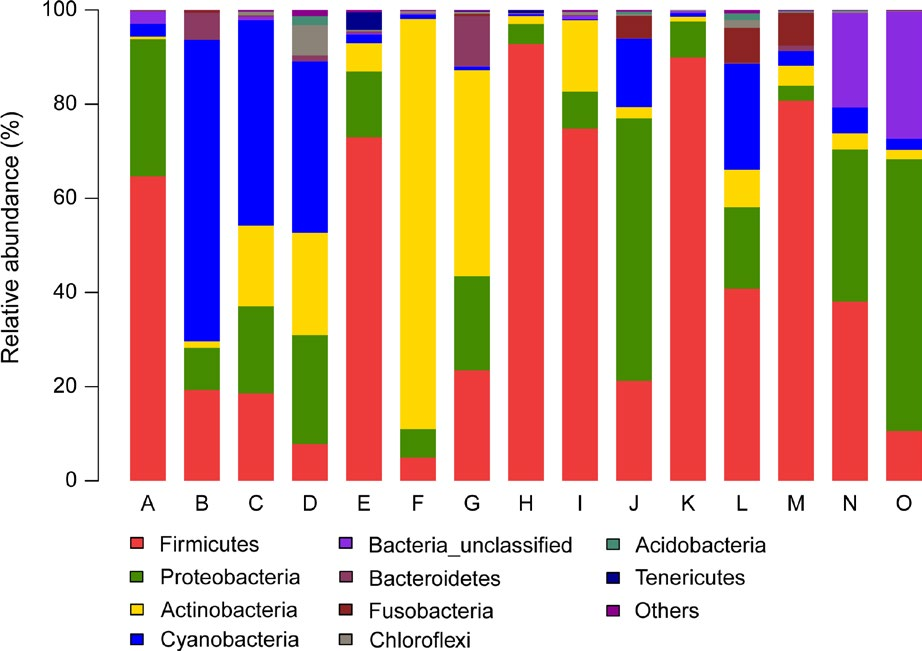
Distribution of phyla among 15 hooded crane fecal samples

We identified 22 different bacterial phyla in ≥1 sample. Figure 3 shows the relative abundance of the gut microbiome of the 15 samples. Five phyla were present in all samples as follows: Firmicutes (average 44%), Proteobacteria (average 20.4%), Actinobacteria (average 14.4%), Cyanobacteria (average 13.3%), and Fusobacteria (average 1.4%). These phyla were abundant in most samples, which represented an average of 93.5% of the microbial communities. Firmicutes were dominant in most samples.

We identified members of 188 families in at least one sample, and 27 families were detected in all samples. The relative distributions of the 10 most abundant core families were as follows: Peptostreptococcaceae (average 13.8%), Enterobacteriaceae (average 11%), Clostridiaceae (average 9.8%), Planococcaceae (average 6.6%), Micrococcaceae (average 4.8%), Microbacteriaceae (average 4%), Lactobacillaceae (average 3.4%), Veillonellaceae (average 2%), Paenibacillaceae (average 1.7%), and Ruminococcaceae (average 1.7%).

**Figure 4:**
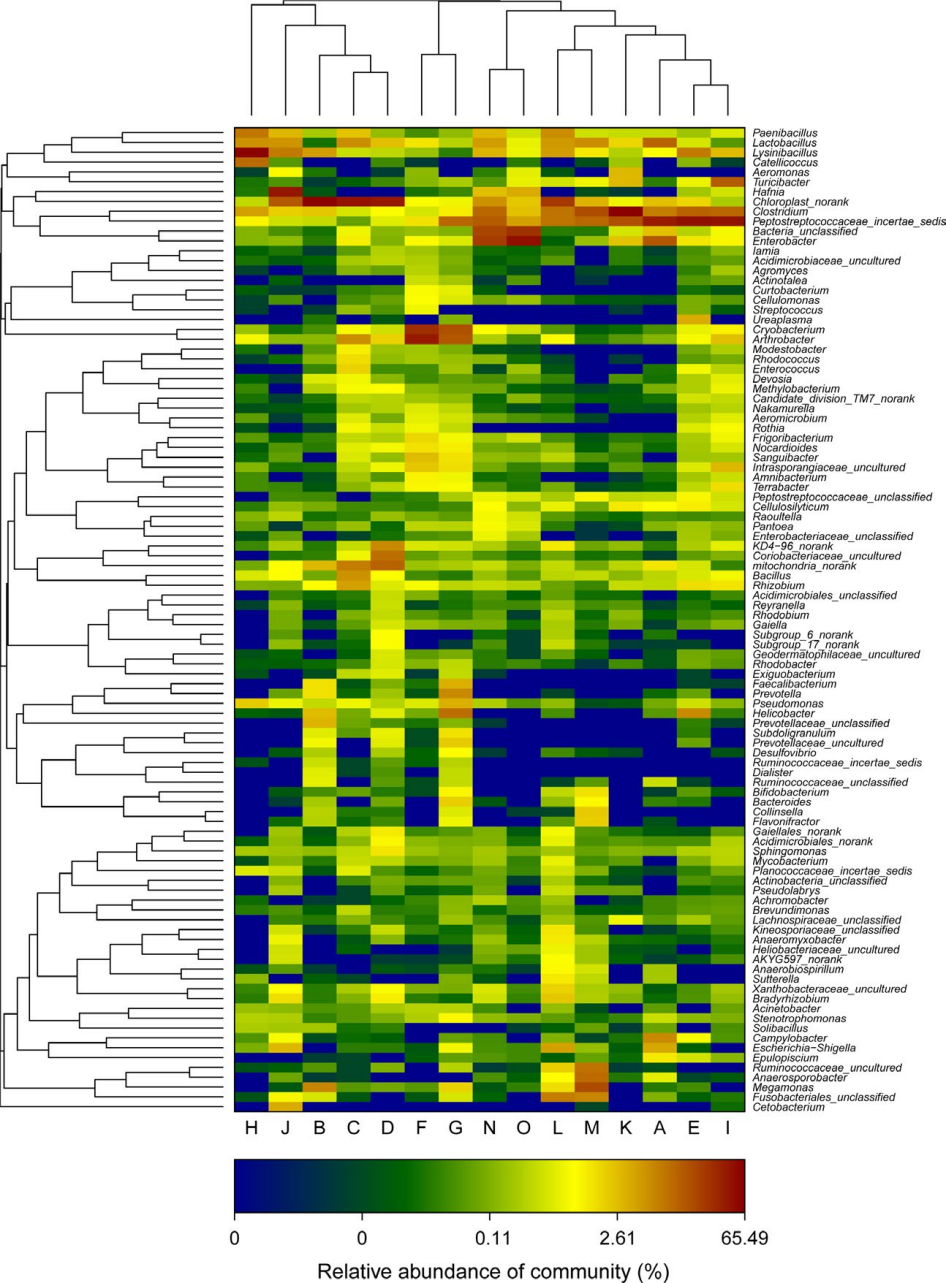
Distribution of the top 100 most abundant genera among the 15 hooded crane fecal samples

According to high‐throughput sequencing results, we identified 305 genera among the 15 fecal samples. *Peptostreptococcaceae* incertae sedis was the most abundant division (*M* = 13.5%) followed by *Chloroplast norank* (*M* = 13.3%). The first and second most frequent genera among the 15 samples were *Clostridium* (*M* = 9.8%) and *Lysinibacillus* (*M* = 6.4%). And the heatmap (Figure 4) of the frequencies of the genera reveals three distinct groups comprising samples B, C, D, H, and J; F and G; A, E, I, M, N, K, L, and O, respectively.

### 3.3 Core gut microbiome

Analysis of the frequencies of genera revealed that 37 genera were present in >90% of the samples and 26 genera were present in all samples, suggesting that approximately 12% of the genera were consistently present among most of the hooded cranes sampled, and that 9% were present at varying levels in all samples. Excluding genera present at <0.1% of the total reads, 20 genera were present in all samples, and 6 genera were present in 14 samples (Tables 2 and 3). Therefore, 9% of the gut microbiome formed the core microbiome of >90% samples. *Peptostreptococcaceae* genera (*M* = 13.5%) were not identified at genus level.

**Table 2:** The relative abundance of core genera (100% core threshold) in the guts of hooded cranes

Genus	% of total	Phylum	Class	Order	Family
*Clostridium*	9.8	Firmicutes	Clostridia	Clostridiales	Clostridiaceae
*Enterobacter*	6.4	Proteobacteria	Gammaproteobacteria	Enterobacteriales	Enterobacteriaceae
*Lysinibacillus*	6.4	Firmicutes	Bacilli	Bacillales	Bacillaceae
*Arthrobacter*	4.5	Actinobacteria	Actinobacteria	Actinomycetales	Micrococcaceae
*Lactobacillus*	3.4	Firmicutes	Bacilli	Lactobacillales	Lactobacillaceae
*Cryobacterium*	3.3	Actinobacteria	Actinobacteria	Actinomycetales	Microbacteriaceae
*Paenibacillus*	1.7	Firmicutes	Bacilli	Bacillales	Paenibacillaceae
*Turicibacter*	1.3	Firmicutes	Bacilli	Bacillales	Turicibacteraceae
*Rhizobium*	0.7	Proteobacteria	Alphaproteobacteria	Rhizobiales	Rhizobiaceae
*Pseudomonas*	0.6	Proteobacteria	Pseudomonadales	Pseudomonadales	Pseudomonadaceae
*Bacillus*	0.7	Firmicutes	Bacilli	Bacillales	Bacillaceae
*Bradyrhizobium*	0.4	Proteobacteria	Alphaproteobacteria	Rhizobiales	Bradyrhizobiaceae
*Cellulosilyticum*	0.4	Firmicutes	Clostridia	Clostridiales	Lachnospiraceae
*Frigoribacterium*	0.3	Actinobacteria	Actinobacteria	Actinomycetales	Microbacteriaceae
*Nocardioides*	0.3	Actinobacteria	Actinobacteria	Actinomycetales	Nocardioidaceae
*Terrabacter*	0.3	Actinobacteria	Actinobacteria	Actinomycetales	Intrasporangiaceae
*Sphingomonas*	0.3	Proteobacteria	Alphaproteobacteria	Sphingomonadales	Sphingomonadaceae
*Stenotrophomonas*	0.2	Proteobacteria	Alphaproteobacteria	Rhizobiales	Xanthomonadaceae
*Raoultella*	0.2	Proteobacteria	Gammaproteobacteria	Enterobacteriales	Enterobacteriaceae
*Pantoea*	0.2	Proteobacteria	Gammaproteobacteria	Enterobacteriales	Enterobacteriaceae

Shown are only genera that represented >0.1% of the total reads. The *Peptostreptococcaceae* incertae sedis (12.6%) were not identified at the genus level.

**Table 3:** The relative abundance of the newly added core genera (90% core threshold) in the guts of hooded cranes

Genus	% of total	Phylum	Class	Order	Family
*Methylobacterium*	0.2	Proteobacteria	Alphaproteobacteria	Rhizobiales	Methylobacteriaceae
*Devosia*	0.2	Proteobacteria	Alphaproteobacteri	Rhizobiales	Hyphomicrobiaceae
*Cellulomonas*	0.1	Actinobacteria	Actinobacteria	Actinomycetales	Cellulomonadaceae
*Mycobacterium*	0.2	Actinobacteria	Actinobacteria	Actinomycetales	Mycobacteriaceae
*Gaiella*	0.1	Actinobacteria	Actinobacteria	Gaiellales	Gaiellaceae
*Nakamurella*	0.1	Actinobacteria	Actinobacteria	Actinomycetales	Nakamurellaceae

Shown are only genera that represented ≥0.1% of the total reads.

## 4 DISCUSSION

The present study is the first to describe the gut microbiome of hooded cranes wintering at Shengjin Lake. The feces of the hooded cranes harbored an abundant population of microbes that included 785 OTUs representing 22 phyla, 51 classes, 107 orders, 188 families, and 305 genera. Further analysis showed that a high diversity of intestinal microbial of hooded crane samples, the Simpson index and Shannon–Wiener index averaged 0.17 and 2.83, respectively. One study indicates that the location of the sampling site mainly reflects the composition of microbiota compared with taxonomy or ecology (Hird et al., 2014). This maybe because Shengjin Lake provides wintering hooded cranes with an abundant and diverse source of food.

The most abundant phyla of gut microbes of wintering hooded cranes were as follows: Firmicutes, Proteobacteria, and Actinobacteria. The microbial content was similar to wild bar‐headed geese: Firmicutes predominated (58.33%), followed by Proteobacteria (30.67%), Actinobacteria (7.33%), and Bacteroidetes (3.33%) (Wang et al., 2016a; Wang et al., 2016b). The most populous phylum was Firmicutes that includes species that catabolize complex carbohydrates, polysaccharides, sugars, and fatty acids to provide an energy source for the host (Flint et al., 2008; Tap et al., 2009). The high abundance of Firmicutes was largely accounted for by *Clostridium*,* Lysinibacillus*, and *Lactobacillus*, which are often found as flora of the giant panda and hoatzin (Godoyvitorino et al., 2012; Zhu et al., 2011). Proteobacteria and Actinobacteria inhabit mammals, including horse (Dougal et al., 2013) as well as avian species such as the bar‐headed goose (*Anser indicus*) (Wang et al., 2016a; Wang et al., 2016b). In the present study, we detected a high abundance of Proteobacteria, mainly *Enterobacter*. The high abundance of Actinobacteria was largely accounted for by *Arthrobacter*. The gut microbiomes of some seabirds such as king penguins harbor a high abundance of Leuconostocaceae, Campylobacteriaceae, Porphyromonadaceae, Helicobacteriaceae, Flavobacteriaceae, Moraxellaceae, and Streptococcaceae (Dewar et al., 2013). Furthermore, Leuconostocaceae and Streptococcaceae are the most abundant families of gut bacteria of the short‐tailed shearwater (Dewar et al., 2014), in contrast to those of hooded cranes.

The microbial composition of the gut of the hooded cranes may be related to their diet acquired from their major foraging habitats such as paddy fields and meadows (Zheng et al., 2015). Further study is required to understand the relationship between the function of “key” bacteria and the lifestyle of hooded cranes, particularly their feeding habits. In addition, some sequences were classified as *Chloroplast norank*, because they likely represent ingested plant material.

We show here that 9% of the genera formed the core gut microbiome of hooded cranes. These core microbes influence and determine the composition of the intestinal microbial community structure and maintain community balance. According to previous studies, we detected many probiotics among the core microbes, which are characterized by remarkable metabolic and physiologic versatility, providing nutrition for the host. For example, cellulolytic *Clostridium* was the most abundant genus (average 9.8% of the total) (Sabathe et al., 2002; Shoham et al., 1999; Varel & Pond, 1992; Warnick et al., 2002) as well as *Bacillus*,* Cellulosilyticum*,* Cellulomonas* that could convert cellulose into metabolites. Furthermore, we detected an abundance of *Arthrobacter* (averaged 4.5% of the total), which as nutritionally versatile bacteria existed in many animals’ gut system (Buchan et al., 2001; Lu & Domingo, 2008). *Lactobacillus* (averaged 3.4% of the total), which was found in all samples, can degrade starch into maltose, maltotriose, and glucose (Champ et al., 1983; Kotarski et al., 1992), and are abundant in the seed‐eating green‐rumped parrotlet (*Forpus passerinus*) (Pacheco et al., 2004). Further studies are required to simultaneously test and verify the diet of the host as one of the factors that influence the composition of the gut microbiota as well as studies to determine the functions of the core gut microbiome to better understand the diet and health of the hooded cranes that winter at Shengjin Lake.

We detected many potential pathogenic bacteria. For example, *Corynebacterium*, which was detected in 13 samples, includes several species that cause disease in mammals and birds (Hoelzle et al., 2013; Potti et al., 2002). *Helicobacter*, an opportunistic pathogen, were detected in 12 samples and are often present in other birds and animals (Oxley & McKay, 2005). *Staphylococcus*,* Streptococcus*, and *Pasteurella* were detected in 14, 8, and 3 samples, respectively. Because hooded cranes migrate annually over long distances to obtain an abundant food supply, they are potentially exposed to novel and pathogenic microbes. Moreover, pressures generated by changing lifestyles and diets associated with migration might disrupt the stable gut microbiota and diminish immunocompetence associated with a corresponding increase in susceptibility to pathogens (Owen & Moore, 2006). Therefore, we were not surprised to find these potentially pathogenic microbes. Further studies must focus on the identification of the species of these potential pathogens as well as their potential to cause disease. In addition, we have detected some zoonotic disease pathogens. For example, the *Campylobacter* detected in 13 samples may cause bacterial gastroenteritis in human (Whelan et al., 1988), and *Escherichia–Shigella*, which were detected in 13 samples, are potential human pathogens that cause diarrhea (Hermes et al., 2009). The *Pseudomonas* found in all samples is opportunistic human pathogens (Chan et al., 2015). There are large flock of poultry and livestock breeding at Shengjin Lake. The habitats of domestic waterfowl overlap those of the hooded cranes at Shengjin Lake, and large aggregations of migratory birds may represent a source of pathogenic microbes that can be transmitted through feces.

## 5 CONCLUSIONS

This study first identified the gut microbiome of hooded cranes and defines the core gut microbiome of hooded cranes wintering at Shengjin Lake. We have shown that the feces of the hooded cranes harbored an abundant population of microbes. The core gut microbiome of hooded cranes wintering at Shengjin Lake includes many probiotics. In addition, we detected many potentially pathogenic bacteria; furthermore, metagenomics and the identification of these potential pathogens should be undertaken. Our study provides a foundation for further studies aimed to characterize the normal digestive functions and health of this endangered species.

## CONFLICT OF INTEREST

None declared.
